# Political Leadership in the Time of Crises: Primum non Nocere

**DOI:** 10.1371/currents.dis.fd8aaf6707cd5dd252e33c771d08b949

**Published:** 2015-05-29

**Authors:** Frederick M. Burkle, Dan Hanfling

**Affiliations:** Harvard Humanitarian Initiative, Harvard University, Cambridge, Massachusetts; The Woodrow Wilson International Center for Scholars, Washington, DC, USA; University of Pittsburgh Medical Center, Center for Health Security, Baltimore, Maryland, USA; Department of Emergency Medicine, George Washington University, Washington, DC, USA

**Keywords:** Ebola, Epidemics, Global health, Science and politics

## Abstract

Long before the 2014 Ebola outbreak in West Africa, the United States was already experiencing a failure of confidence between politicians and scientists, primarily focused on differences of opinion on climate extremes. This ongoing clash has culminated in an environment where politicians most often no longer listen to scientists. Importation of Ebola virus to the United States prompted an immediate political fervor over travel bans, sealing off borders and disputes over the reliability of both quarantine and treatment protocol. This demonstrated that evidenced- based scientific discourse risks taking a back seat to political hyperbole and fear. The role of public health and medical expertise should be to ensure that cogent response strategies, based upon good science and accumulated knowledge and experience, are put in place to help inform the development of sound public policy. But in times of crisis, such reasoned expertise and experience are too often overlooked in favor of the partisan press “sound bite”, where fear and insecurity have proved to be severely counterproductive. While scientists recognize that science cannot be entirely apolitical, the lessons from the impact of Ebola on political discourse shows that there is need for stronger engagement of the scientific community in crafting messages required for response to such events. This includes the creation of moral and ethical standards for the press, politicians and scientists, a partnership of confidence between the three that does not now exist and an “elected officials” toolbox that helps to translate scientific evidence and experience into readily acceptable policy and public communication.

## Introduction

“…the scary thing is that I want a leader who consults experts and thinks about all of the different sides to an issue before making statements and policies that are unfounded in science.”

Kaci Hickox, RN a quarantined nurse, February 2015

A failure in the timing and content of the expression of risk, both immediate and long term, has always proved a critical misstep in communicating to the public. As the medical crisis appears to be coming to a tenuous closure in West Africa it is time to reflect on the response to Ebola in the United States which unfortunately resulted in a manufactured social and political crisis. It shouldn’t be this way, yet an exploration of numerous critical events over the past quarter century demonstrate that time and time again, politics trumps science. Unfortunately, the promotion of evidenced based decision making has too often taken a back seat to policies based upon fear and insecurity. And the great facilitator, the 24 hour news cycle, has been there to fuel the fire of fear as the American public contemplates the risks and threats of a disease that at the time of this writing has only resulted in the import of a handful of cases. Humanitarian healthcare workers and a journalist have been repatriated for evaluation and management in the United States, one imported case from the epicenter in Liberia made its way to Dallas, Texas, unrecognized in its initial presentation, and two nosocomial infections transmitted to a poorly prepared healthcare workforce occurred as a result. In fact, more people will be injured or killed on the nation’s highways in the time it takes to read these opening comments than have been affected by the Ebola virus disease (EVD) in the U.S.

The Ebola epidemic was not the first time that US federal and state issues have clashed over ownership to respond with authority to an infectious disease emergency. In fact, during the 2003 SARS pandemic it was argued by states that possession of the legal responsibilities, including quarantine, under the US Constitution belonged to them rather than the federal government.^1^ Yet today, laws within states vary widely in defining what criteria establishes a quarantine. This includes five states in which CDC has unique authority to issue a mandatory quarantine at ports of entry---but only if the infectious agent is on the list of diseases specified under Executive Order of the President.^2^^,^ 3 In most cases in which the clinical expression of infectious disease is concerned, uncertainty often precedes definitive diagnosis. Reliance upon basic clinical laboratory efforts that take time to achieve definitive diagnostic analysis is the norm. Yet, there is often a rush to make crucial public decisions despite incomplete evidence, placing responsibility for defining these risks to the public health on political actors within state and local governments.

The political climate that enveloped the U.S. polity, where mistrust in government runs high, and partisan accusations rule the airwaves, made the complexity of the Ebola response all the more difficult to explain to the American public. As the epidemic mushroomed in West Africa, the front line responders from Medicin sans Frontieres raised and waved their arms and called upon the global community to pay attention to the fact that Ebola had gone urban where the greater density of the population would contribute to its rapid spread. It became increasingly clear the outbreak was very much out of control. WHO was slow to react and political attention in the U.S. was focused upon other competing national security priorities. Over the summer of 2014, the shooting war in Ukraine, Syria and the Gaza crisis, reminiscent of the ‘spy versus spy’ political instability of the cold war era, easily bested a medical crisis developing in a part of the world that has only know war, famine, and political upheaval. How could Ebola be of concern here? In the absence of a firm grasp of what constitutes the global health security agenda, political leaders were unable to fathom the consequences of an emerging infectious disease spreading out of control. And they were not ‘managed up’ by those medical leaders in positions of authority to do so. When the first two US humanitarian healthcare workers who contracted EVD were repatriated to the U.S., the framing of a cogent dialogue related to the management of Ebola were instead replaced by abject fear and concern magnified out of proportion to the risk. In the absence of a process that can be used to help political leaders navigate the complexities involved in the evaluation of medical, public health and scientific concerns, discussion revolved around the emotions of response, not the science. Because most political leaders have no grounding in scientific inquiry and are unable to recite even basic tenets related to public health and medical response, Ebola was addressed as if it were the latest terrorist faction to emerge seeking to disrupt civil society.

Examination of previous events that have been characterized by epidemics of fear share a number of key attributes. First, these claims that fuel fear have no weight. They are always driven by the media. They occur because the voice of science is overtaken by the voice of fear. In the case of the Ebola crisis, neither the voice of politics nor the media have provided any alternative science that adequately counters observable evidence or the risks facing the nation. Unfortunately, this most often occurs because rapid evolution of policy based on available and changing evidence makes it difficult for the “message” to keep up with the facts. In the gap between what is known and what is needed to know lies the opportunity for fear to dictate the messaging and, therefore, policy.

Since the second half of the 20th century, “the world has seen the relationship between society, politics and science become increasingly complex and controversial.”^4^ A decade of climate change debate has exemplified this building frustration. There is no lack of studies in health sciences or medicine that have shown that making information useful demands engagement with those who will use it.^5^ Shamefully, the political climate, both in Europe and the U.S., has not allowed this to happen; allowing “politicians, influential intellectuals and lobbyists who oppose research and innovation for various reasons”, which include political prejudices and religious arguments, to adopt “a strategy of manipulating and censoring facts.”^4^ In fact, the more politicians ignore science the more it represents a failure of governance .^6^

President George W. Bush upset the previously guarded political independence of science and politics by establishing controversial key appointments and science and health policies that went against expert advice^4^. Today, politicians listen to economists not to scientists. 6 Scientists must be impartial arbiters of evidence based data, they cannot join politics, be political agents or compete as lobbyists.^6^^,^ 7 Unfortunately, many politicians, when challenged with views of the science, too often lead with harsh and glib retorts that prove beneficial when facing uninformed electorates. Indeed, it has been recognized that the poorly debated Ebola controversies influenced the 2014 U.S. mid-term elections.

The unsettling irony is that while “public prestige of science is higher than ever it remains disturbingly removed from the centers of power.”^8^ Nine out of ten scientists believe that political parties have adopted an “anti-science stance on issues ranging from evolution to climate change”.^9^ While this unproductive competition has smoldered unnoticed by the public over the past decade, the Ebola crisis and the outright dismissal of science by political decision makers has revealed today the absolute depths of the conflict. Worse, while politicians readily engage on a ‘one sound bite’ level on Sunday morning television they quickly refuse to debate the science…or flippantly state they’ll “leave that to the scientists to explain” while leaving the strong impression that the scientists and practitioners of that science need to do a better job.

## The Political and Scientific Challenges of Public Health Emergencies

Reflex charges of incompetence of public health experts and unfounded disagreements over proven health management protocols have resulted in delayed interventions. Scientific input and their evidence base has been marginalized or discounted in favor of partisan interest. This is nothing new. The reliance on and incorporation of scientific framing of the public health issues related to the Deepwater Horizon oil spill 10 and the Fukushima Daichi nuclear disaster 11 were similarly cast in the context of fear, not evidence. Hysteria generated over the return of asymptomatic healthcare workers from Ebola endemic regions to stigma and isolation forced on them by a poorly informed political class are just the latest examples. In the case of the debate over quarantine, the inability to develop consensus on this issue across the U.S. Federal interagency, with Department of Defense personnel being subject to forced 21 day quarantine, exacerbated the tremendous uncertainty mirrored by public confusion.

In an increasingly globalized world, scientists recognize that health alone will not solve what demands multidisciplinary and interdisciplinary solutions. The emerging global health discipline requires “composite research” of many disciplines for which health is but one.^12^ Current political discourse, however, tends to favor single discipline concrete answers that are contrary to more insightful, reflective and abstract thinking that decision making skills of diplomacy typically relies on. Macilwain cautions that while “everyone knows that the most valuable work is now multidisciplinary”, the U.S. Congress, during a critical time when the social sciences would benefit them most in improving their “public engagement” and image, have “expelled” social sciences research from their agenda.^8^

Kassen contends that poor scientific decisions in politics is primarily a “failure of scientists to communicate their message effectively in what is ultimately a political, not a scientific, arena.” 13 Recognizing that it is the role of journalists to “ask the tough question”, the authors acknowledge that politicians become vulnerable and susceptible when such questions are no more than a convenient trapdoor designed more to measure the mettle of the politician through a challenging and often unanswerable query than as a test of their immediate knowledge of the politician’s grasp of the science. It is during the palpable silence between the question and the answer that scientists experience the most anxiety. Scientists do not know what information the politicians have, from what source the information originates (e.g., CDC, NIH, military) or how solid the information is before they speak with the authority of their office. In defense of the politician, we remind the readers that “the practice of science cannot be, nor should it be, entirely apolitical.” 14 The rush of anxiety among scientists occurs when there is violation of the principle of separation of science and politics that “first we need the science, then the politics.” 14

## What Needs to Occur?

Politicians should be prepared to define health and public health entities and be able to explain them to a broad public audience who look to them as both leaders and educators. Predictably, politicians are expected to explain complex situations in a manner understood by the public at-large, reinforce that information during press conferences and bring in experts at the proper time. Particularly given the complexity involved in those situations in which the science unfolds over time, such events ought to be slowed down and can be better managed. The public deserves it and the politicians should demand it. Once misinformation is blurted out the scientific community is often pressed to correct, recover and reason the confusion in facts… not a position that most scientists relish or are readily prepared to do especially when the science is still not known or well understood. Accountability and transparency is expected; the public is more trusting and less suspicious of information that is admitted to be either unknown or still being studied.

Recognizing the impact of rapid advances in communications and social media, the most desirable outcome of the Ebola tragedy, or any crises that has the potential to be or has already been declared a public health emergency, is that politicians, journalists and scientists must address, debate and develop a working framework, including the adoption of moral and ethical standards, that are mutually defined and agreed upon as partners in bringing the proper information to the public in a timely and effective manner. The bottom line is that all “scientists need to be able to negotiate with governments, irrespective of their political hue, and to advise politicians in a useful and timely way.” 6 Politicians do not read scientific journals 5 nor are likely to do so even during a health crisis, when the knowledge-base is imperfect.^15^ Britain, in particular, “loves its scientific advisors", boasts that "almost every government department has one" and the central government “in turn, looks to their Chief Scientific Advisor to enhance the credibility of their policies. ” 8 Favaro sees that “scientific-liaison offices” would give scientists an apolitical route to policy formation. This would make research results accessible and enable politicians and policy makers to reach informed decisions 6 and further allow politicians to benefit politically from emphasizing the importance of communicating the value of that science to the public.

Scientists and practitioners must recognize that politicians will always possess a bully pulpit access to the public that they themselves will never have.^16^ For example, work by a multidisciplinary panel of scientists, health practitioners and legal scholars on crisis standards of care 17 ^,^ 18 ^,^ 19 and the promotion of health care emergency operations 20 were able to highlight the importance of promoting involvement of the highest levels of elected office, in part by promoting the development of targeted risk communications strategies. It is increasingly evident that such efforts must include the direct engagement of elected officials in the crafting and delivery of crisis communications, particularly when complicated health and medical issues may be part of the core message. This can take the form of an “elected official’s toolkit” developed to promote understanding of basic health and medical concepts, and should serve to translate science into policy in a manner in which the political agenda can be supported, not subverted, by fact and evidence.

Indeed, in February 2015 the (US) Presidential Commission for the Study of Bioethical Issues, in making recommendations for the next epidemic, concluded that the nation must improve its health infrastructure, emergency response and be ready to respond quickly. The Commission urged that ethics expertise be part of the planning. Among the controversial issues that were politically charged, the Commission stated that a single US health official be placed in charge and that health officials communicate often and clearly explaining the “rationale behind health policies to a frightened public.” “Travel curbs and quarantine must be based on science and use the least restrictive means necessary”, emphasizing that “needlessly restricting the freedom of expert and caring health workers is both morally wrong and counterproductive.^21^ Craig Spencer, a physician who developed Ebola after returning to the US, wrote that “politicians should have educated the public about the science of Ebola and acted accordingly.”^22^

The Ebola crisis has become a teachable moment in the nexus between politics and science. Experts and advisors in scientific knowledge, methods and substance can provide lessons emanating from this current crisis that will be of tremendous benefit in future events. We must first teach our political leaders what has been a fundamental medical precept of Hippocrates, that of* primum non nocere*, first do no harm.

## Competing Interests

The authors have declared that no competing interests exist.
